# Lipid disorders in children living with overweight and obesity- large cohort study from Poland

**DOI:** 10.1186/s12944-020-01218-6

**Published:** 2020-03-16

**Authors:** Michał Brzeziński, Paulina Metelska, Małgorzata Myśliwiec, Agnieszka Szlagatys-Sidorkiewicz

**Affiliations:** 1 Department of Public Health and Social Medicine, Medical University of Gdańsk, al. Zwycięstwa 42a, 80-210 Gdańsk, Poland; 2 “6-10-14 for Health” University Clinical Center, ul. Dębinki 7, 80-952 Gdańsk, Poland; 3 Department of Pediatrics, Diabetology and Endocrinology, Medical University of Gdańsk, Debinki 7, 80-952 Gdańsk, Poland; 4 Department of Paediatrics, Gastroenterology, Allergology & Paediatric Nutrition, Medical University of Gdansk, ul. Nowe Ogrody 1-6, 80-803 Gdansk, Poland

**Keywords:** Metabolic syndrome, Dyslipidemia, Pediatric obesity

## Abstract

**Background:**

While in the general paediatric population the presence of abnormal lipid values is estimated at 8–20%, depending on the population, accepted norms and age, it was shown that in the population of lean children the prevalence of dyslipidemia is lower than in obese children, in whom it ranges from 20 to over 40%. Until now, however, no results of similar studies on a large sample of children form a Central or Eastern European country have been published. The aim of this study was to evaluate the prevalence of lipid disorders in overweight and obese children and adolescents participating in an integrated weight reduction programme.

**Methods:**

According to the “6-10-14 for Health” programme implementation schedule, the programme accepted patients living in Gdańsk, aged 6, 9–11 and 14 years old, with BMI above the 85th percentile for age and sex, according to the Polish percentile charts. During the first visit, each of the participants underwent basic anthropometric examinations - body weight, body height, waist and hip circumference, blood pressure and body composition by bioelectrical impedance were measured. Blood samples were taken to assess lipid, glucose and insulin levels as well as alanine transaminase (ALT) and thyroid stimulating hormone (TSH) activity.

**Results:**

1948 patients underwent full anthropomethric and blood work measurements. At least one of the lipid disorders occurred in 38.23% of girls and 40.51% of boys with overweight and obesity. The most common lipid disorderswere decreased high-density lipoprotein cholesterol (HDL-C) levels (present in 20.55% of the girls and 23.79% of the boys) and elevated low-density lipoprotein cholesterol (LDL-C) (present in 15.31% of the girls and 14.25% of the boys). There was no strong association between lipid disorders and age, sex, birth weight, gestational age at birth or body composition.

**Conclusions:**

Such a frequent occurrence of lipid disorders in the population of children and adolescents should be an important warning signal both at the individual and population level. Not only effective screening methods for overweight and obese children should be implemented from an early age but also therapeutic measures are required.

**Trial registration:**

The trial is registered under the Local Ethics Committee at Medical University of Gdańsk, decision No. NKBBN/228/2012 from 25 June 2012.

## Background

The prevalence of overweight and obesity in the child and adolescent population is a growing problem worldwide, with the number of obese children rising from 0.7 and 0.9% in 1976 to 5.6 and 7.8% in 2016 for girls and boys respectively [1]. Although the rate of this increase varies by country, it also applies to Central and Eastern Europe [2]. Data on the high percentage of children with extreme obesity, reaching even as much as 5.5% in some populations, is even more worrying [3]. Excess body weight is a significant burden for the current and future health and mortality risk of children, with issues including type 2 diabetes mellitus, dyslipidemia, non-alcoholic fatty liver disease, hypertension, and coronary heart disease as well as psychological problems and lower educational attainment seen as obesity consequence in paediatric population [4–6]. Long-term observations have shown that obesity in childhood and adolescence may significantly contribute to the occurrence of further disorders such as glucose metabolism disorders, cardiovascular diseases and lipid disorders [7–10]. This can lead to a reduction in the quality and length of life [11].

While in the general paediatric population the presence of abnormal lipid values is estimated at 8–20% [12], depending on the population, accepted norms and age, it was shown that in the population of lean children the prevalence of dyslipidemia is lower than in obese children, in whom it ranges from 20% to over 40% [8, 13]. As Nielsen et al. demonstrated, the risk of developing lipid disorders is 2.8 times higher in obese children (BMI > 90th percentile) than in children with normal body weight [13].

Co-occurrence of excess body weight and lipid disorders was assessed in many populations, including studies carried out in China, Brazil, Denmark, Germany, USA, Ghana, United Arab Emirates [12–17] . Until now, however, no results have been published of similar studies on a large sample of children form a Central or Eastern European country, in which a rapid change of social and economic status of society in the last 30 years has been observed, [18].

The aim of this study was to evaluate the prevalence of lipid disorders in overweight and obese children and adolescents participating in an integrated weight reduction programme. The analysis reported in this paper was carried out based on data from a population-based health programme aimed at preventing the occurrence of risk factors for civilization diseases in children and adolescents “6-10-14 for Health”, implemented by the University Clinical Centre in Gdańsk using funds from the Municipal Office in Gdańsk between 2011 and now.

## Methods

According to the “6-10-14 for Health” programme implementation schedule [19, 20], the programme accepted patients living in Gdańsk, aged 6, 9–11 and 14 years old, with BMI above the 85th percentile for age and sex, according to the Polish percentile charts [21]. During the first visit, each of the participants underwent basic anthropometric examinations - body weight, body height, waist and hip circumference, blood pressure and body composition by bioelectrical impedance were measured.

### Body weight and body height

were determined using a digital scale (MensorWE150, Poland), with the child wearing underwear and standing barefoot. Body height was measured to the nearest 0.001 m and body weight to the nearest 0.1 kg. The scale was calibrated every day. Waist and hip circumferences were measured on a horizontal plane by an Ergonomic Circumference Measuring Tape (model 201; Seca GmbH & Co, KG, Hamburg, Germany).

### Blood pressure measurement

Arterial blood pressure was determined oscillometrically (Omron) on the left arm, with a cuff of an adequate size placed at the heart level, and the child seated with uncrossed legs, following at least a 5-min rest in the seated position. The width of the inflatable cuff corresponded to at least 40% of arm circumference. Three separate measurements of blood pressure were taken and averaged.

### Kasch pulse recovery step test

The participants were subjected to a 3-min Kasch pulse recovery step test (KPR). The test consisted of climbing a 0.305 m step at a rate of 24 up-and-down steps per minute. The rate of climbing was defined by a metronome set at 96 beats (signals) per minute. Heart rate (HR) was monitored continuously with he “Polar” (Finland) electronic analyser for 3 min of the exercise (step-test) and during 1 min and 5 s of recovery in a seated position. Only post-exercise HR recorded within 1 min, starting 5 s after completing the test, was analysed. All HR characteristics were recorded during restitution in a seated position (subjects were instructed to sit still, breathe normally and not to talk). An arithmetic mean calculated from these values (HRmean post-ex) was subjected to a further analysis [22].

Additionally, on the basis of data from parents (based on the child health logbook), information on body weight and gestational age at birth was collected. Information was also collected on the current body weight and height of the child’s parents, on the basis of which the parents’ BMI at the time of the child’s examination was subsequently calculated.

### Laboratory parameters

All enrolled children were referred for laboratory tests within a maximum of 30 days following the first appointment in the programme. After an overnight fast, venous blood samples were drawn between 7 and 9 a.m., processed within 1 h, and analysed within 6–8 h after sampling at the Central Clinical Laboratory of the University Clinical Centre (UCC) in Gdańsk. The following laboratory parameters were determined:

- Lipid profile determined using an enzymatic method.
- Oral glucose tolerance test (OGTT) using glucose concentration determined with hexokinase method.
- Concentration of insulin determined by means of immunochemiluminescence assay.
- Concentration of creatinine, alanine transaminase (ALT), thyroid stimulating hormone (TSH), free thyroxine (fT4) levels determined using an immunoturbidimetric assay.

Dyslipidemia was defined according to the Lipid Research Clinical (LRC) Prevalence Study and the United States National Health and Nutrition Examination Surveys (NHANES) classification corresponding to the 95th percentile in the American population as total cholesterol (TC) > 5.2 mmol/L (200 mg/dL), LDL-C > 3.4 mmol/L (130 mg/dL), HDL-C < 1.03 mmol/L (40 mg/dL), or triglycerides (TG) > 1.7 mmol/L (150 mg/dL) [23, 24].

The study was conducted with the approval of an independent bioethics committee at the Medical University of Gdańsk (NKBBN/228/2012.), in accordance with the requirements of the Helsinki Declaration. Each parent/legal guardian had to express written consent to the child’s participation in the programme.

### Statystical analysis

BMI was calculated and then referred to the age- and sex-specific national BMI percentile charts. The 85th percentile of BMI was used as the overweight cut-off [21]. Z-score values of the waist circumference were determined for the relevant age and sex group using current percentile charts for Poland [25]. Z-score values for systolic blood preasure (SBP) and diastolic blood preasure (DBP) were determined for the relevant age and sex group using current percentile charts for Poland [26]. To assess the normality of distribution in individual groups, Shapiro-Wilk test was used, with appropriate parametric tests subsequently used (t-test, ANOVA, Pearson correlation) for data with normal distribution and non-parametric tests used for data with distribution different from normal (U Mann-Whitney, Kruskal-Wallis test with post hoc analysis, Chi-square, Spearman rank correlations). Data are presented as means and standard deviations (SD) or numbers and valid percentages. Statistical significance was accepted at the *p* < 0.05 level. Only the data from patients whounderwent all laboratory tests were included in the analysis. Statistical analyses were performed using Statistica software version 13.3 (StatSoft Polska Sp. z o.o., Poland).

## Results

In the years 2011–2017, according to the adopted criteria (children in the 1st and 4-6th grade of primary school and the 2nd grade of junior high school in Gdańsk), just under 70,000 children were eligible for the study. Over 46,000 children took part in the screening, of which 5005 children were qualified for the programme. The first appointment was attended by 3062 (61.18%) children, of which 1948 children underwent the laboratory tests they were referred for during the first visit, allowing for the assessment of the prevalence of lipid disorders, metabolism of glucose disorders and hormonal management disorders to be carried out within 30 days from the examination.

**Fig. 1:**
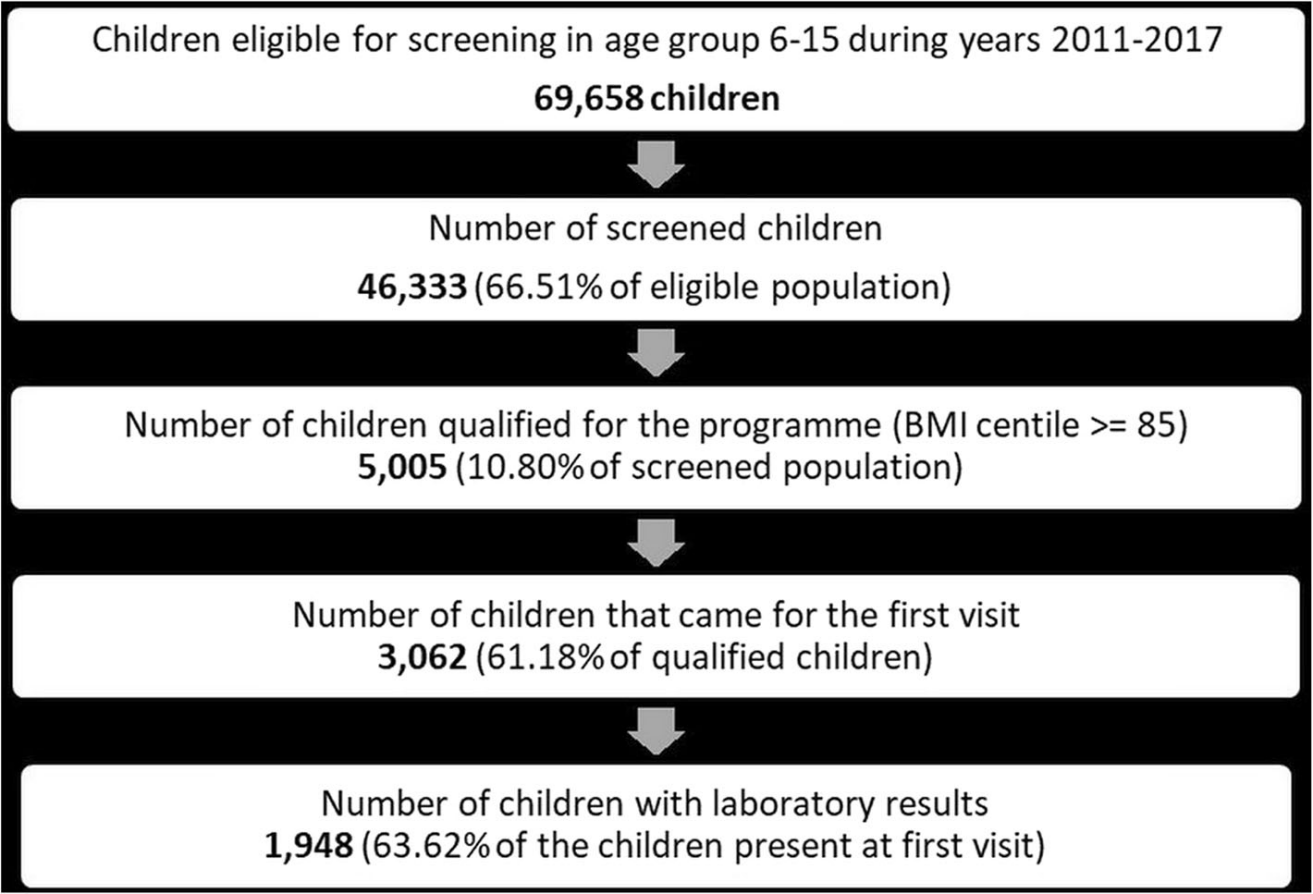
Number of children participating in the successive stages of the “6-10-14 for Health” programme during years 2011–2017

A detailed description of patient flow is presented in Fig. 1.

Table 1 presents characteristics of children who participated in the first appointment and did and did not undergo laboratory tests. There were statistically significant differences between these groups concerning their BMI percentile, waist z-score and the percentage of girls. The group that had the laboratory tests performed had a higher proportion of girls, a higher BMI percentile and a higher waist z-score.

**Table 1:** Difference in basic anthropometry results in children with and without laboratory results

	with laboratory results			without laboratory results	
	No. valid	Mean ± SD	No. valid	Mean ± SD	*p*-value
age (years)	1916	10.66 ± 3.01	1040	10.74 ± 3.17	0.663
BMI percentile	1916	93.97 ± 5.44	1040	92.54 ± 7.88	**0.000**
gestational age (weeks)	1858	40.02 ± 4.39	998	39.86 ± 3.89	0.439
weight at birth (grams)	1862	3492.89 ± 666.70	990	3490.89 ± 650.93	0.610
father’s BMI	1802	28.00 ± 8.02	926	27.55 ± 8.30	0.090
mother’s BMI	1865	27.26 ± 5.15	985	28.86 ± 4.11	0.261
waist z-score	1915	1.67 ± 0.60	1039	1.60 ± 0.67	**0.003**
SBP z-score	1916	0.61 ± 1.16	1040	0.70 ± 1.20	0.058
DBP z-score	1916	1.05 ± 1.18	1040	1.10 ± 1.26	0.418
% of girls	49.94		44.60		0.004*

U Man-Whitney test, *p*-value was bolded as statistically significant if *p* < 0.05.; * Chi^2 Pearson’s test

In children with laboratory tests’ results, several statistically significant differences in anthropometric values were observed. Boys had lower BMI percentile (93.6 vs. 94.3), lower body fat percentage (26.63 vs. 33.01) and higher values of lean body mass (73.33 vs. 66.94). Boys also had higher body weight at birth (3591 g vs. 3470 g) (*p* < 0.05). There were no clinically significant differences in lipid values in both studied groups (except for a slight difference in triglyceride levelsbetween boys and girls at 82.89 vs. 83.83 respectively). Details are presented in Table 2.

**Table 2:** Differences in selected anthropometric features and lipid profile values between girls and boys in the study group

Variable	Girls			Boys	
	No. valid	Mean ± SD	No. valid	Mean ± SD	*p*-value
age (years)	956	10.64 ± 3.07	960	10.68 ± 2.95	0.569
weight at birth (grams)	917	3470.93 ± 536.04	924	3591.78 ± 582.25	**0.000**
gestational age (weeks)	923	39.79 ± 1.80	927	39.73 ± 1.79	0.302
father’s BMI	847	29.50 ± 4.66	861	29.58 ± 4.80	0.962
mother’s BMI	909	26.69 ± 5.14	907	26.59 ± 5.18	0.648
BMI percentile	956	94.33 ± 5.22	960	93.61 ± 5.62	**0.000**
waist z-score	956	1.39 ± 0.56	959	1.95 ± 0.49	**0.000**
bioimp_fat_mass_%	951	33.01 ± 5.87	955	26.63 ± 6.06	**0.000**
bioimp_muscle_mass_%	951	63.64 ± 4.75	955	69.59 ± 5.53	**0.000**
bioimp_lean_mass _%	951	66.94 ± 6.03	954	73.33 ± 6.51	**0.000**
SBP z-score	956	0.57 ± 1.18	960	0.65 ± 1.14	0.143
DBP z-score	956	1.11 ± 1.22	960	1.00 ± 1.13	0.064
TC (mg/dL)	973	169.44 ± 28.68	975	167.14 ± 29.61	0.098
HDL (mg/dL)	973	47.39 ± 9.75	975	47.10 ± 10.61	0.285
TG (mg/dL)	973	83.83 ± 40.19	975	82.89 ± 46.98	**0.013**
LDL (mg/dL)	973	105.28 ± 25.28	975	103.46 ± 26.28	0.197

U Man-Whitney test, *p*-value was bolded as statistically significant if *p* < 0.05

The percentage of children with particular types of lipid disorders is presented in Table 3. It was shown that the only difference between the sexes concerned the abnormal triglyceride levels, which were more common in boys (8.82% vs. 5.96%).

**Table 3:** Number and percentage of children with particular types of disorders in lipid profile

	TC > 200 mg/dL	LDL-C > 130 mg/dL	HDL-C < 40 mg/dL	TG > 150 mg/dL
Total	278 (14.27%)	288 (14.78%)	432 (22.17%)	144 (7.39%)
Girls	142 (14.49%)	149 (15.31%)	200 (20.55%)	58 (5.96%)*
Boys	136 (13.94%)	139 (14.25%)	232 (23.79%)	86 (8.82%)*

* *p* = 0.015 Chi^2 Pearson’s test

At least one of the above lipid disorders occurred in 38.23% of girls and 40.51% of boys with overweight and obesity. The difference was not statistically significant (*p* = 0.30297 Chi^2 Person’s test).

The results of laboratory tests were subsequently classified into six categories following the model used by Korsten-Reck et al. [27].

A: Normolipidemia [triglycerides (TG) < 150 mg/dL, HDL-C (HDL-C) > 40 mg/dL];
B: Hyper-LDL-cholesterolemia alone [LDL-C > 130 mg/dL, TG < 150 mg/dL];
C: Hypo-HDL-C + Hypertriglyceridemia [HDL-C < 40 mg/dL, TG > 150 mg/dL];
D: Combined hyperlipidemia = Hyper-LDL-C + Hypertriglyceridemia [LDL-C > 130 mg/dL, TG > 150 mg/dL]; with added two additional categories for the clarity of classification of disorders:
E: Hypercholesterolemia only [TC > 200 mg/dL];
F: Other disorders (TG > 200 mg/dLor HDL-C < 40 mg/dL or HDL-C < 40 mg/dL + LDL-C > 130 mg/dL/TG > 150 mg/dL).

The prevalence rates for particular groups of disorders are presented in Table 4. There were no sex differences in the prevalence of lipids disorders (*p* = 0.171 Chi-square Person’s test).

**Table 4:** Number and percentage of children with specific lipid disorders

Group	Total		Girls		Boys	
	No.	valid %	No.	valid %	No.	valid
**A** Normolipidemia	1181	60.63	601	61.77	580	59.49
**B** Hyper-LDL-C	264	13.55	138	14.18	126	12.92
**C** Hypo-HDL-C+ Hyper-TG	59	3.03	20	2.06	39	4.00
**D** Hyper-LDL-C + Hyper-TG	24	1.23	11	1.13	13	1.33
**E** Hyper-TC	60	3.08	30	3.08	30	3.08
**F** (Other - in text above)	360	18.48	173	17.78	187	19.18

*p* = 0.171 Chi-square Person’s test

When analysing the results for children with at least one disorder as one group, compared to the group of children without disorders, we found that children with dyslipidemia were older and had higher values of basic anthropometric parameters such as BMI percentile, percentage of body fat or waist z-score. Details are presented in Table 5.

**Table 5:** Differences between overweight and obese children with and without dyslipidemia

	dyslipidemia	normolipidemia	
	Mean ± SD	Mean ± SD	*p*-value
age (years)	10.9 ± 2.96	10.5 ± 3.03	**0.022**
BMI percentile	94.5 ± 4.67	93.6 ± 5.86	**0.000**
bioimp_fat_mass_%	30.4 ± 7.41	29.4 ± 6.29	**0.006**
bioimp_muscle_mass_%	66.1 ± 6.11	67.0 ± 5.83	**0.005**
bioimp_lean_mass_%	69.5 ± 7.05	70.5 ± 7.01	**0.003**
weight at birth (grams)	3501.9 ± 535.72	3550.9 ± 579.20	**0.033**
gestational age (weeks)	39.7 ± 1.79	39.8 ± 1.80	0.153
father’s BMI	29.8 ± 4.77	29.4 ± 4.70	0.091
mother’s BMI	26.6 ± 5.22	26.6 ± 5.12	0.906
waist z-score	1.8 ± 0.61	1.6 ± 0.57	**0.000**
DBP z-score	1.1 ± 1.15	1.0 ± 1.19	0.084
SBP z-score	0.7 ± 1.15	0.6 ± 1.16	**0.025**

U Man-Whitney test, *p*-value was bolded as statistically significant if *p* < 0.05

Further analysis showed a correlation between individual anthropometric data and the results of laboratory tests. The analysis demonstrated a statistically significant (*p* < 0.05) correlation between numerous parameters, however, the strength of the correlation was not high - except for bioimpedance data. Detailed results for Spearman’s correlation are presented in the Table 6.

**Table 6:** Spearman rank correlations between selected anthropometric parameters and individual lipid profile values

	TC	HDL-C	TG	LDL-C
age (years)	**−0.179**	**−0.238**	**0.200**	**−0.166**
BMI percentile	−0.033	**−0.154**	**0.139**	−0.021
bioimp_fat_mass_%	0.035	**− 0.083**	**0.142**	0.035
bioimp_muscle_mass_%	−0.040	**0.079**	**−0.144**	− 0.038
bioimp_lean_mass_%	−0.037	**0.086**	**−0.146**	− 0.036
weight at birth (grams)	−0.012	**0.054**	**−0.089**	− 0.014
gestational age (weeks)	−0.035	0.026	−0.022	− **0.051**
father’s BMI	0.001	**−0.072**	**0.058**	0.023
mother’s BMI	**−0.057**	**−0.050**	0.019	**−0.050**
waist z-score	**−0.070**	**−0.229**	**0.129**	−0.040
SBP z-score	−0.033	**−0.092**	**0.131**	−0.041
DBP z-score	−0.004	**−0.080**	**0.111**	−0.007

Spearman correlation, *p*-value was bolded as statistically significant if *p* < 0.05

## Discussion

According to the American Academy of Pediatrics (AAP), National Heart, Lung and Blood Institute (NHLBI) and other recommendations, children with obesity or familial aggregation of hyperlipidemia should have their lipid concentrations monitored regularly [28, 29]. However, in Poland such tests are not conducted regularly or financed by the public payer (tests are included in the PHC package, but there are no guidelines requiring such tests to be performed in children).

The results presented in this paper for the first time describe the prevalence of lipid disorders in a large group of overweight and obese children and adolescents from a Central and Eastern European country. The obtained results show that lipid disorders in children aged 6–15 years are more frequent than expected in comparison with populations from other European countries. In a Danish study Nielsen found lipid disorders in 28% of overweight and obese children in a similar age group [13]. The results are similar to those obtained in American studies (concerning children from all ethnic groups) [30] and a German study analysing children from a clinic specializing in the treatment of obesity [12].

At least one type of a lipid disorder occurred in more than 39% of the study group, while in the current German and Danish studies these values were 24,7 and 28% respectively. In the current study group there were no significant differences in the prevalence of particular lipid disorders between boys and girls, except for elevated TG values, which were more common in girls, as described in other studies [12, 13], but not in all of them [15]. Differences in the prevalence of specific lipid disorders by sex are not commonly reported, although previously mentioned study by Nielsen et al. [13] showed that the lipids level were higher in girls – in all ages. This was also reported by Jolliffe et al. [23] based on US NHANES population. There is also a visible effect of puberty,with a decrease in HDL-C, LDL-C and TC and an increase in TG in pubertal compared to prepubertal children – as seen in both the general population and in obese children [13]. As we did not assess the pubertal status of our population we cannot compare that factor with other studies. Age itself was not a factor significantly differentiating the group,although there were negative correlations between age and lipid values, however, these correlations were weak...

Children with lipid disorders were older, had higher values of BMI percentile, body fat percentage and waist circumference, which is consistent with the previously published results for studies carried out in Europe and on other continents [12–15]. However, it appears that the anthropometric values, as presented in Table 5, do not have a significant clinical utility. Differences in mean age/ BMI or bioimpedancy values are not big. We need to remember that those are all children with overweight and obesity. Presented correlations despite their statistical significance, do not indicate a strong (positive or negative) relationship between different lipids with the measured anthropometric parameters, or with birth weight and gestational age at birth. According to current guidelines [31], only children with severe obesity (>99th BMI centile) should be treated as a “moderate-risk” group. Children with obesity (<99th BMI centile) are “at risk” of cardiovascular disease – regardless of other risk factors that were not assessed in the presented study (ex. non-alcoholic fatty liver disease, hypertension) as the group was otherwise healthy (no patients had diagnosed familiar hyperlipidemia, T1/2 diabetes mellitus, cancer survivors). In the studied population both children with and without lipid disorders had mean BMI below the 99 centile (94.5 ± 4.67 vs. 93.6 ± 5.86).

It should be noted that the obtained results concerning the prevalence of particular types of lipid disorders may indicate the prevalence of combined dyslipidemia in more than 20% of the examined children, with an additional 13% of children having elevated LDL-C values. The atherogenicity of the combined dyslipidemia observed with childhood obesity manifests in structural and functional vascular changes assessed non-invasively as increased carotid intima-media thickness (cIMT) and increased arterial stiffness [32]. Data from *Cardiovascular Risk in Young Finns Study* showed that subjects with the combined dyslipidemia pattern beginning in childhood had significantly increased cIMT compared with normolipidemic controls after 21 years of follow up [33]. Yet, as results from adults show, it seems that the low HDL-C or high LDL-C levels can’t be seen as the only measures of cardiovascular risk. Studies assessing the genetic differences determining lipid profile (both LDL-C and HDL-C levels) show that genetic background can be an important factor for the future outcome of low or elevated levels of blood lipids [34, 35]. This show us that a long term effects of elevated or decreased lipid level can only be treated as one element in a wider range of sociodemographic issues – lifestyle, nutritional and physical activity behaviours that can influence the CVD risk through the adolescence and adult life.

Such a frequent occurrence of lipid disorders in the population of children and adolescents should be an important warning signal both at individual and population level. Not only effective screening methods should be implemented for overweight and obese children from an early age [36] but therapeutic measures are also required. It has been repeatedly demonstrated that implementing proper nutrition and/or physical activity significantly improves the lipid profile, even without significant weight loss (we do not strive for this in younger children), and mainly by changing the body fat percentage [32]. Additionally, children at medium and high risk of developing CVD should also receive proper pharmacological treatment [31].

The results of our study showed that despite the lack of a significant upward trend in the prevalence of obesity in the population of children in Poland [1, 37, 38], lipid disorders are a common problem in this group of children and may significantly contribute to the increase in cardiovascular risk in the population of young adults [39]. It is important to state that Poland has undergone significant social and economic changes in the last 30 years that have impacted both the development of the country and health status of the population – including large increases in the prevalence of obesity and related cardiovascular diseases rates in adults [39, 40]. Until now no data were published regarding dyslipidaemia as an important CVD risk factors in children.

The study has certain limitations. Despite the high population coverage and participation of over 66% of children in the screening, only about 60% of children who met the eligibility criteria joined the programme, and subsequently only less than 64% had laboratory tests performed. There is a justified risk that children who did not participate in the programme, despite meeting the eligibility criteria, had higher values of anthropometric parameters or already existing metabolic disorders. Similarly, children who did not have laboratory tests performed - despite the lack of statistically significant differences in anthropometric parameters (Table 1) - could have had lipid disorders, although our analysis shows that they did not differ statistically (analysis in progress) in terms of anthropometric features from the group included in the final analysis.

## Conclusions

Our study presents results from a large group of real life patients participating in an obesity management programme. Almost 40% of the study population had lipid abnormalities. Most importantly, children with dyslipidemia were older and had higher values of basic anthropometric parameters such as BMI percentile, percentage of body fat or waist z-score than the overweight and obese children without lipid disorders. The study shows that the probability of lipid disorders is higher in children with a higher body mass and age irrespectively of gestational age and weight or parents’ body mass.

## Data Availability

The datasets used and/or analysed during the current study are available from the corresponding author on reasonable request.

## Abbreviations

ALT: Alanine transaminase
BMI: Body mass index
cIMT: Carotid intima-media thickness
CVD: Cardiovascular diseases
DBP: Diastolic blood pressure
fT4: Free thyroxine
HR: Heart rate
KPR: Kasch pulse recovery step test
OGTT: Oral glucose tolerance test
SBP: Systolic blood pressure
TC: Total cholesterol
TG: Triglycerides
TSH: Thyroid stimulating hormone

## References

[1] Bentham J, Di Cesare M, Bilano V, Bixby H, Zhou B, Stevens GA (2017). Worldwide trends in body-mass index, underweight, overweight, and obesity from 1975 to 2016: a pooled analysis of 2416 population-based measurement studies in 128·9 million children, adolescents, and adults. Lancet.

[2] Ahrens W, Pigeot I, Pohlabeln H, De Henauw S, Lissner L, Molnár D (2014). Prevalence of overweight and obesity in European children below the age of 10. Int J Obes.

[3] Spinelli A, Buoncristiano M, Kovacs VA, Yngve A, Spiroski I, Obreja G (2019). Prevalence of Severe Obesity among Primary School Children in 21 European Countries. Obes Facts Karger Publishers.

[4] Park MH, Falconer C, Viner RM, Kinra S (2012). The impact of childhood obesity on morbidity and mortality in adulthood: a systematic review. Obes Rev.

[5] Rankin J, Matthews L, Cobley S, Han A, Sanders R, Wiltshire HD (2016). Psychological consequences of childhood obesity: psychiatric comorbidity and prevention. Adolesc Health Med Ther.

[6] Bhadoria A, Sahoo K, Sahoo B, Choudhury A, Sufi N, Kumar R (2015). Childhood obesity: causes and consequences. J Fam Med Prim Care Medknow.

[7] Freedman DS, Khan LK, Serdula MK, Dietz WH, Srinivasan SR, Berenson GS (2005). The relation of childhood BMI to adult adiposity: the Bogalusa heart study. Pediatrics.

[8] Umer A, Kelley GA, Cottrell LE, Giacobbi P, Innes KE, Lilly CL (2017). Childhood obesity and adult cardiovascular disease risk factors: a systematic review with meta-analysis. BMC Public Health.

[9] Lloyd LJ, Langley-Evans SC, McMullen S (2010). Childhood obesity and adult cardiovascular disease risk: a systematic review. Int J Obes.

[10] Callo Quinte G, Barros F, Gigante DP, de Oliveira IO, dos Santos Motta JV, Horta BL (2019). Overweight trajectory and cardio metabolic risk factors in young adults. BMC Pediatr.

[11] Dietz WH (1998). Childhood weight affects adult morbidity and mortality. J Nutr Narnia.

[12] Dathan-Stumpf A, Vogel M, Hiemisch A, Thiery J, Burkhardt R, Kratzsch J (2016). Pediatric reference data of serum lipids and prevalence of dyslipidemia: results from a population-based cohort in Germany. Clin Biochem.

[13] Nielsen TRH, Lausten-Thomsen U, Fonvig CE, et al. Dyslipidemia and reference values for fasting plasma lipid concentrations in Danish/North-European White children and adolescents. BMC Pediatr. 2017;17:116. 10.1186/s12887-017-0868-y. 10.1186/s12887-017-0868-y PMC5410076 28454530

[14] Song Peige, Yu Jinyue, Chang Xinlei, Wang Manli, An Lin (2017). Prevalence and Correlates of Metabolic Syndrome in Chinese Children: The China Health and Nutrition Survey. Nutrients.

[15] Lartey A, Marquis GS, Aryeetey R, et al. Lipid profile and dyslipidemia among school-age children in urban Ghana. BMC Public Health. 2018;18:320. 10.1186/s12889-018-5196-0. 10.1186/s12889-018-5196-0 PMC5840796 29510714

[16] Haroun D, Mechli R, Sahuri R, et al. Metabolic syndrome among adolescents in Dubai, United Arab Emirates, is attributable to the high prevalence of low HDL levels: a cross-sectional study. BMC Public Health. 2018;18(1):1284. 10.1186/s12889-018-6215-x. 10.1186/s12889-018-6215-x PMC6249919 30463538

[17] Reuter CP, Tatiana P, Dagmar J, Renner P, De Mello ED, RDM V (2015). Original article dyslipidemia is associated with unfit and overweight-obese children and adolescents.

[18] Hlavaty P, Tvrzicka E, Stankova B, Zamrazilova H (2015). Association of plasma lipids fatty acid composition with metabolic profile of Czech adolescents.

[19] Brzezinski M, Jankowski M, Niedzielska A, Danielewicz A, Czarnecka P (2014). Health program &quot;6-10-14 for Health&quot;• as an example of comprehensive environmental activities in the field of children obesity. Study protocol and primary results. Appetite.

[20] Szlagatys-Sidorkiewicz A, Brzeziński M, Jankowska A, Metelska P, Słomińska-Fraczek M, Socha P (2017). Long-term effects of vitamin D supplementation in vitamin D deficient obese children participating in an integrated weight-loss programme (a double-blind placebo-controlled study) - rationale for the study design. BMC Pediatr.

[21] Kułaga Z, Litwin M, Tkaczyk M, Palczewska I, Zaja̧czkowska M, Zwolińska D (2011). Polish 2010 growth references for school-aged children and adolescents. Eur J Pediatr.

[22] Jankowski M, Niedzielska A, Brzezinski M, Drabik J (2015). Cardiorespiratory fitness in children: a simple screening test for population studies. Pediatr Cardiol Springer.

[23] Jolliffe CJ, Janssen I (2006). Distribution of lipoproteins by age and gender in adolescents. Circulation.

[24] Articles S (2011). Expert panel on integrated guidelines for cardiovascular health and risk reduction in children and adolescents : summary report. Pediatrics.

[25] Kułaga Z, Litwin M, Małgorzata Zajączkowska M, Wasilewska A, Morawiec-Knysak A, Różdżyńska A (2008). Comparison of waist and hip circumferences ranges in children and adolescents in Poland 7–18 y of age with cardiovascular risk thresholds – initial results of OLAF project (PL0080). Stand Med.

[26] Kułaga Z, Litwin M, Grajda A, Kułaga K, Gurzkowska B, Góźdź M (2012). Oscillometric blood pressure percentiles for Polish normal-weight school-aged children and adolescents. J Hypertens.

[27] Korsten Katrin (2008). Frequency of secondary dyslipidemia in obese children. Vascular Health and Risk Management.

[28] Zachariah JP, Johnson PK (2014). Pediatric lipid management: an earlier approach. Endocrinol Metab Clin North Am NIH Public Access.

[29] Daniels SR, Greer FR, Committee on Nutrition (2008). Lipid screening and cardiovascular health in childhood. Pediatrics.

[30] Kit BK, Kuklina E, Carroll MD, Ostchega Y, Freedman DS, Ogden CL (2015). Prevalence of and trends in dyslipidemia and blood pressure among US children and adolescents, 1999-2012. JAMA Pediatr American Medical Association.

[31] De Ferranti SD, Steinberger J, Ameduri R, Baker A, Gooding H, Kelly AS (2019). e603 On behalf of the American Heart Association Athero-sclerosis, Hypertension and Obesity in the Young Committee of the Council on Cardiovascular Disease in the Young; Council on Cardiovascular Radiology and Intervention; Council on Cardiovascular and Stroke Nursing; Council on Clinical Cardiology; and Council on Quality of Care and Outcomes Research Cardiovascular Risk Reduction in High-Risk Pediatric Patients Circulation. Circulation.

[32] Cook Stephen, Kavey Rae Ellen W. (2011). Dyslipidemia and Pediatric Obesity. Pediatric Clinics of North America.

[33] Juonala M, Viikari JSA, Rönnemaa T, Marniemi J, Jula A, Loo B-M (2008). Associations of dyslipidemias from childhood to adulthood with Carotid Intima-Media thickness, elasticity, and Brachial Flow-Mediated dilatation in adulthood. Arterioscler Thromb Vasc Biol.

[34] Voight BF, Peloso GM, Orho-Melander M, Frikke-Schmidt R, Barbalic M, Jensen MK (2012). Plasma HDL cholesterol and risk of myocardial infarction: a mendelian randomisation study. Lancet Lancet Publishing Group.

[35] Bull CJ, Bonilla C, Holly JMP, Perks CM, Davies N, Haycock P (2016). Blood lipids and prostate cancer: a Mendelian randomization analysis. Cancer Med Blackwell Publishing Ltd.

[36] Expert Panel on Integrated Guidelines for Cardiovascular Health and Risk Reduction in Children and Adolescents, National Heart, Lung, and Blood Institute (2011). Expert panel on integrated guidelines for cardiovascular health and risk reduction in children and adolescents: summary report. Pediatrics.

[37] Brzeziński M, Jankowski M, Jankowska A, Niedzielska A, Kamińska B (2018). Is there a rapid increase in prevalence of obesity in Polish children? An 18-year prospective observational study in Gdansk, Poland. Arch Med Sci.

[38] Kułaga Z, Grajda A, Gurzkowska B, Wojtyło M, Góźdź M, Litwin M. The prevalence of overweight and obesity among Polish school- aged children and adolescents. Przegl Epidemiol. 70:641–51 [cited 2018 Aug 5] Available from: http://www.ncbi.nlm.nih.gov/pubmed/28233966. 28233966

[39] Stepaniak U, Micek A, Waśkiewicz A, Bielecki W, Drygas W, Janion M (2016). Prevalence of general and abdominal obesity and overweight among adults in Poland Results of the WOBASZ II study (2013–2014) and comparison with the WOBASZ study (2003–2005). Pol Arch Med Wewn.

[40] Rutkowski M, Bandosz P, Czupryniak L, Gaciong Z, Solnica B, Jasiel-Wojculewicz H (2014). Prevalence of diabetes and impaired fasting glucose in Poland-the NATPOL 2011 Study. Diabet Med.

